# Current Evidence for Combined Targeted Therapy for the Treatment of Inflammatory Bowel Disease

**DOI:** 10.1093/jcag/gwad032

**Published:** 2023-09-26

**Authors:** Panu Wetwittayakhlang, Peter L Lakatos

**Affiliations:** Division of Gastroenterology and Hepatology, McGill University Health Center, Montreal, Quebec, 1650 Cedar Ave, Montreal, QC, H3G 1A4, Canada; Gastroenterology and Hepatology Unit, Division of Internal Medicine, Faculty of Medicine, Prince of Songkla University, 15 Karnjanavanich Road, Hat Yai, Songkhla, 90110, Thailand; Division of Gastroenterology and Hepatology, McGill University Health Center, Montreal, Quebec, 1650 Cedar Ave, Montreal, QC, H3G 1A4, Canada; Department of Oncology and Medicine, Semmelweis University, 1083, Korányi Sándor u. 2/a, Budapest H-1085, Hungary

**Keywords:** inflammatory bowel disease, combination therapy, biologic, combined targeted therapy

## Abstract

Biologicals and small molecules have revolutionized the medical management of inflammatory bowel diseases (IBD), yet they are only effective in a proportion of patients, and their impact on changing the natural history of the disease is still debatable. Recently, the concept of combining targeted biologics and small-molecule therapies has been introduced to the treatment of IBD. Dual-targeted therapy (sequential and combined), which is the combination of two targeted therapies, might be a reasonable choice for patients to break through the therapeutic ceiling. A recent randomized clinical trial (VEGA) provided the first controlled evidence that the short-term combination of two biological agents may lead to superior disease control than either of the agents alone in patients with ulcerative colitis (UC) without jeopardizing safety. Multiple studies are underway in both Crohn’s disease and UC. Additionally, real-world evidence is accumulating in IBD patients receiving combination therapies with concomitant IBD and extraintestinal manifestations or in patients with medically refractory IBD. Of note, the majority of these patients were exposed to multiple biological agents earlier and lost response to at least one of the agents in the combination. This review summarizes current knowledge regarding this attractive novel therapeutic option in IBD. Clearly, more controlled data are needed to evaluate optimal timing, efficacy, and mitigation of safety concerns.

## Concept of combined targeted therapy in treatment of inflammatory bowel disease

Biological therapies have become the standard of care for moderate to severely active inflammatory bowel disease (IBD), including Crohn’s disease (CD), and ulcerative colitis (UC).^1,2^ However, the current treatments for IBD achieve control in only two-thirds of users at best.^3^ With the available biologic and small-molecule therapies, overall clinical remission rates are at best 50%. Rates of achieving remission with anti-tumor necrosis factor (anti-TNF) therapy for the induction of remission were reported in 18% to 48% of CD patients.^4^ In UC, a meta-analysis comparing gut-selective anti-integrin α4β7, vedolizumab with anti-TNFs showed that the pooled rates of mucosal healing were similar and varied from 18% to 33% following 32 to 54 weeks of maintenance treatment.^5^ Similarly, in the VARSITY study, the rate of clinical remission at 52 weeks was low, at only 31.3% and 22.5% for vedolizumab and adalimumab, respectively.^6^ The efficacy of a Janus kinase inhibitor, tofacitinib for induction of clinical remission at 8 weeks in UC patients was 16.6% to 18.5%, and remission rates at 52 weeks ranged from 34.3% to 40.6% in the initial responders.^7^ Of note, approximately 50% of patients who initially responded to biological therapy eventually lose their response over time.^8,9^ As a result, IBD patients who are refractory or have lost their response to a certain biologic therapy need to switch to a different molecule, reducing their treatment options.

The rationale for combining targeted therapy has been initially explored in rheumatologic and dermatologic diseases. Multiple randomized controlled trials (RCTs) have failed to show additional benefit for combination of biological therapy (e.g., etanercept and abatacept, anakinra and etanercept, or rituximab and atacicept) compared to mono-biologic therapy to achieve superior disease control.^10^ Furthermore, rheumatologic patients receiving combined biologic therapy had a higher frequency of adverse events compared to patients receiving biologic monotherapy.^10,11^ The majority of the RCTs in the rheumatology literature, however, included biologics that are not approved for the treatment of IBD. Thus, extrapolating data from the rheumatologic literature to the treatment of IBD is not possible because agents approved for the treatment of IBD were not included in rheumatologic studies.

The concept of combined targeted therapy has been proposed in the management of IBD. Given that multiple inflammatory pathways are simultaneously activated in the intestinal mucosa, blocking only one of them may not be enough to optimal control inflammation in each patient. Monotherapy with a biologic agent that is effective for luminal disease may not be as effective for controlling co-existing extraintestinal manifestations (EIMs) or other immune-mediated inflammatory diseases (IMIDs). Therefore, combined targeted therapy with different mechanisms of action appears to be a reasonable strategy for refractory IBD patients who failed or lost response to a single biological agent or who have active co-existing EIMs or IMIDs. In this review, we discuss the current evidence for combined biological and/or small-molecule therapies for the treatment of IBD.

## Current evidence of combined targeted therapy in IBD

### Randomised controlled trials and clinical trials

The summarized RCTs in combined targeted therapy for IBD are shown in Table 1.

**Table 1.** The RCT studies with combined targeted therapy in IBD.

Authors (year)	Patient	Treatment	Finding
Sands et al. (2007)^12^	79 active CD inadequate response to anti-TNF	IFX + natalizumab (52) vs. IFX + placebo (27)	Clinical remission at week 2, 6 and 10 was 15.4%, 23.1%, and 36.5%, respectively Higher decrease in mean CDAI score in IFX + natalizumab group compared to IFX+ placebo, but not significant.
Columbel et al. EXPLORER trial (2022)^13^	55 biologic naïve patients with high-risk CD	VDZ + ADA + MTX	Clinical remission 54.4% Endoscopic remission 34.5%
Feagan et al. VEGA study (2023)^14^	214 severe active UC, anti-TNF naive	GUS + GOL (71) vs. GUS (71) or GOL (72) monotherapy	At week 12 Clinical response: GUS+GOL (83%) vs. GUS (75%) vs. GOL (61%) Clinical remission: GUS +GOL (37%) vs. GUS (21%) vs. GOL (22%) Endoscopic improvement: GUS+GOL (49%) vs. GUS (25%) vs. GOL (30%) Endoscopic remission: GUS+GOL (18%) vs. GUS (8%) vs. GOL (10%) At week 38 of maintenance treatment: Clinical response: GUS+GOL (69%) GUS (72%) vs. GOL (58%) Clinical remission: GUS+GOL (44%) GUS (31%) vs. GOL (22%) Endoscopic improvement: GUS+GOL (49%) vs. GUS (32%) vs. GOL (22%) Endoscopic remission: GUS+GOL (25%) vs. GUS (15%) vs. GOL (7%)
DUET-CD and DUET-UC trial (ongoing phase 2 RCTs)^15,16^	Expected 715 Moderate to severe active refractory CD Expected 550 moderate to severe active refractory UC	GUS, GOL, JNJ-78934804	The estimated primary completion date is July 29, 2024 for DUET-CD (NCT05242471) and August 28,2024 (NCT05242484)

*Abbreviations:* ADA, adalimumab; CD, Crohn’s disease; GOL, golimumab; GUS, guselkumab; IFX, infliximab; MTX, methotrexate; TNF, tumour necrosis factor; UC, ulcerative colitis; VDZ, vedolizumab.

#### Anti-integrin α4 antibody (natalizumab) plus infliximab

This was the first RCT to evaluate the safety and efficacy of the combination of natalizumab and infliximab in patients with active CD despite ongoing infliximab treatment.^12^ In this study of 79 CD patients (52 receiving natalizumab plus infliximab and 27 receiving placebo plus infliximab), the patients receiving combination therapy showed a trend to have higher rates of clinical remission throughout 32 weeks of follow-up compared with patients receiving infliximab monotherapy, but these differences were not statistically significant. However, there were concerns about the safety of natalizumab due to the possibility of progressive multifocal leukoencephalopathy, especially in patients who have had prior immunosuppressive treatment.^17,18^ Due to this, the use of natalizumab in CD treatment has been extremely low and limited to the United States only.^19^

#### The open-label phase 4

“EXPLORER trial,” assessed the efficacy of the triple combination of vedolizumab, adalimumab, and methotrexate therapy in biologic-naive patients with high-risk CD. An interim analysis was undertaken of 55 patients treated with triple therapy (vedolizumab 300 mg IV on day 1 and week 2, week 6 and then every 8 weeks, adalimumab 160 mg SC on day 2, 80 mg at week 2, and then 40 mg every 2 weeks until week 26, methotrexate 15 mg orally weekly until week 34); after the triple therapy and by week 34, every patient received vedolizumab monotherapy until week 102. At week 26, endoscopic response and endoscopic remission were observed in 54.4% and 34.5%, respectively. There were no safety signals related to the triple therapy.^13^

#### The VEGA study

The most recent ongoing phase 2a RCT, evaluated the efficacy of a combination induction therapy with selective interleukin (IL)-23, guselkumab and anti-TNF, golimumab compared to guselkumab or golimumab monotherapy in patients with moderately to severely active UC.^14^ 214 patients naive to an anti-TNF and refractory or intolerant to conventional therapy were randomly assigned to receive guselkumab 200 mg IV at weeks 0, 4, and 8 (*n* = 71); golimumab 200 mg subcutaneous (SC) at week 0, then 100 mg SC at weeks 2, 6, and 10 (*n* = 72); or combination with guselkumab 200 mg IV plus golimumab 200 mg SC at week 0, golimumab 100 mg SC at weeks 2, 6, and 10, and guselkumab 200 mg IV at weeks 4 and 8 (*n* = 71). In the maintenance phase, patients in the combination therapy arm were switched to guselkumab monotherapy at the beginning of week 12.^14^

During the induction, at week 12, 59 (83%) of 71 patients in the combination therapy group had clinical response compared with 44 (61%) of 72 patients in the golimumab monotherapy group (adjusted treatment difference 22.1%; 80% CI 12.9–31.3; *P* = 0.0032) and 53 (75%) of 71 patients in the guselkumab monotherapy group (adjusted treatment difference 8.5%; 80% CI −0.2–17.1; *P* = 0.2155). However, statistical significance was not achieved between the combination therapy group and both monotherapy groups. Similarly, 26 (37%) of 71 patients in the combination therapy group had achieved clinical remission compared with 16 (22%) of 72 patients in the golimumab monotherapy group (*P* = 0.0578) and 15 (21%) of 71 patients in the guselkumab monotherapy group (*P* = 0.0412). In addition, the proportion of patients who had achieved endoscopic improvement (49% vs. 25% vs. 30%) and endoscopic remission (18% vs. 10% vs. 8%) was significantly higher in the combination therapy group than either the golimumab or guselkumab monotherapy groups, respectively. In the maintenance phase, the clinical response and remission rates were largely sustained with guselkumab maintenance in the group that initially received combination therapy. At week 38, 49 (69%) of 71 patients in the combination therapy group had clinical response compared to 42 (58%) of 72 patients in the golimumab monotherapy group (adjusted treatment difference 10.8%; 80% confidence interval, (CI) 1.1–20.5) and 51 (72%) of 71 patients in the guselkumab monotherapy group (adjusted treatment difference −2.8%; 80% CI −11.9–8.3). Furthermore, 31 (44%) of 71 patients in the combination therapy group had achieved clinical remission compared to 16 (22%) of 72 patients in the golimumab monotherapy group (adjusted treatment difference 21.5%; 80% CI 11.9–31.2) and 22 (31%) of 71 patients in the guselkumab monotherapy group (adjusted treatment difference 12.7%; 80% CI 2.7 to 22.7). The proportion of patients who achieved endoscopic improvement (49% vs. 22% vs. 32%) and endoscopic remission (25% vs. 7% vs. 15%) in the combination group was higher compared to both the golimumab monotherapy and guselkumab monotherapy groups, respectively. Adverse event rates were comparable among the treatment groups.^14^

#### JNJ-78934804 (combination of guselkumab and golimumab)

An ongoing phase 2b randomized, double-blind, placebo-controlled trial in patients with moderate-to-severe active CD (DUET-CD trial) and moderate-to-severe active UC (DUET-UC trial). The patients are being randomized into six arms, including guselkumab, golimumab, and JNJ-78934804 (combination guselkumab and golimumab, high-dose, mid-dose, and low-dose) and a placebo arm. The primary outcome of this study is to compare clinical remission and endoscopic response at week 48 among the treatment arms. Both studies are recruiting patients. ClinicalTrials.gov identifiers: NCT05242471 (DUET-CD) and NCT05242484 (DUET-UC).^15,16^

### Real-world evidence

Evidence to support a combination biological treatment strategy is also accumulating from everyday practice. However, one of the main problems with the real-world evidence studies is that the patients were already exposed or lost response to one or both agents used as combination therapy. The summary of the main findings of selected real-world studies on combined targeted therapy in IBD are shown in Table 2.

**Table 2.** The selected retrospective studies in combined targeted therapy in IBD.

Authors (year)	Patient characteristics	Treatment	Main findings
L.Goessens et al. (2021)^20^	58 CD and 40 UC (104 Combination therapy) 70 active IBD 23 active IMID/EIM 10 Both active IBD and active IMID/EIM	41 Anti-TNF + VDZ 11 Anti-TNF + UST 21 UST + VDZ 1 TOF + anti-TNF 13 TOF + VDZ 1 Anti-IL + anti-IL 16 combinations with other molecules	70% clinically improved in IBD activity; complete (26%) or partial improvement (44%) 81% clinical improved in IMID/EIM activity
McShane et al. (2023)^21^	65 CD, 27 UC	33 VDZ + UST 32 VDZ + anti-TNF 17 UST + anti-TNF 12 VDZ + TOF 6 UST + TOF 3 TOF + anti-TNF	46% clinical response at 3 months 34% clinical response at 6 months
Glassner et al. (2020)^22^	31 CD, 18 UC, 1 IBD-U	25 UST + VDZ 8 VDZ + TOF 4 IFX + TOF 4 GOL + TOF 3 UST + TOF 3 ADA + VDZ 2 GOL + VDZ 2 CRZ + VDZ 1 CRZ + TOF 1 ADA + APR	50% clinical remission 65% reduction in steroid use 34% endoscopic remission
Alayo et al. (2021)^23^	25 UC, 10 CD	24 TOF + VDZ 6 TOF + IFX 5 TOF+ UST	At week 8: 50% clinical response 35.7% corticosteroid-free clinical response, 10.7% corticosteroid-free clinical remission At week 16: 66.7% clinical response 58.3% corticosteroid-free clinical response, 37.5% corticosteroid-free clinical remission
Yang et al. (2020)^24^	22 CD	8 UST + VDZ 6 IFX + VDZ 2 ADA + UST 2 CRZ + VDZ 1 ADA + VDZ 1 GOL + VDZ 1 IFX + UST	43% endoscopic improvement 26% endoscopic remission 50% clinical response 41% clinical remission
Lee et al. (2022)^25^	19 CD (active luminal 13, pyoderma gangrenosum 3)	11 TOF +UST 7 TOF + VDZ 1 TOF +CRZ	80.0% clinical response 60.0% clinical remission 54.5% Endoscopic improvement 18.2% endoscopic remission
Privitera et al. (2021)^26^	11 CD, 5 UC	3 VDZ+UST 3 VDZ +ADA 2 UST+IFX 2 VDZ+CRZ 2 VDZ+SKM 1 UST+ADA 1 UST+CRZ 1 VDZ +IFX 1 VDZ+APR	At 6 months, Intestinal symptoms: 42.8% (3/7) clinical response and 14.2% (1/7) remission EIM symptoms: 22% (2/9) clinical response and 55.5% (5/9) remission
Kwapisz et al. (2021)^27^	14 CD, 1 UC	5 UST + VDZ 2 IFX + VDZ 2 ADA + VDZ 3 GOL + VDZ 1 CRZ + VDZ 1 ADA + UST 1 GOL + UST	73% clinical response 67% reduction in steroid use 44% endoscopic or radiological improvement 20% required surgical intervention.

Abbreviations: ADA, adalimumab; APR, apremilast; CD, Crohn’s disease; CRZ, certolizumab; EIMs, extraintestinal manifestations; ETN, etanercept; GOL, golimumab; IBD, inflammatory bowel disease; IBD-U, undetermined inflammatory bowel disease; IFX, infliximab; MS, multiple sclerosis; Pso, psoriatic disease; SKM, secukinumab; SpA, spondylarthritis; TNF, tumor necrosis factor; TOF, tofacitinib; UC, ulcerative colitis; UST, ustekinumab; VDZ, vedolizumab.

A retrospective study by Glassner et al.^22^ evaluated 50 patients with IBD who were treated with various combinations of biologics or small-molecule therapy; approximately 50% were vedolizumab plus anti-interleukin (IL)-12 and IL-23 (ustekinumab) for persistent disease activity (*n* = 47) or concomitant rheumatological or dermatological disease (*n* = 3). There were significantly more patients in clinical remission at 4 months (50% vs. 14%, *P* = 0.0018) and endoscopic remission at 8 months (34% vs. 6%, *P* = 0.0039) compared to baseline.^22^

Another case series revealed that dual biologic therapy was safe and effective in 22 patients with severe refractory CD who had a total of 24 dual biologic treatments after multiple failed biologics. Seven different combinations of biologics were evaluated, including traditional anti-TNF agents (infliximab, adalimumab, golimumab, or certolizumab pegol) combined with vedolizumab or ustekinumab. Clinical response and clinical remission were seen in 50% and 41% of patients, respectively. Endoscopic improvement and remission were found in 43% and 26%, respectively. The presence of active perianal fistula decreased from 50% at baseline to 33% after treatment.^26^ In another case series of 16 patients who received various dual biologic therapies for uncontrolled luminal disease (*n* = 7) or uncontrolled EIMs despite inactive IBD (*n* = 9), the most frequently used combinations were vedolizumab plus ustekinumab and vedolizumab plus adalimumab. A clinical response was reported in all study patients.^20^

In 2021, the most recent large European multicentre retrospective study by Goessens et al.,^21^ reported 98 patients (104 combinations) who started combination therapy for active IBD (67%), active IMID or EIMs (22%), or both (10%), in the setting of multiple biologic failures. The median duration of combination therapy was 8 months (interquartile range, (IQR) 5–16). IBD disease activity was clinically improved in 70% of patients, and IMID/EIM activity was clinically improved in 81%.^23^ A recent multicentre retrospective study, reported on 92 patients receiving combined biologic therapy for active IBD or EIMs. The most common combinations were vedolizumab and ustekinumab (32%), or vedolizumab and anti-TNF (31%). The clinical response rates at 3 and 6 months were 46% and 34%, respectively.^21^

The efficacy of combining tofacitinib with other biological therapies was evaluated in two retrospective cohorts. Alayo et al.^23^ reported that 35 patients (25 with UC and 10 with CD) were started on combination therapy due to a lack of response to their current biologic, despite dose optimization. The most common biologics combined with tofacitinib were vedolizumab (69%), infliximab (17.1%), and ustekinumab (14.3%). At week 8, 50% achieved clinical response, and 35% achieved endoscopic or radiographic remission.^23^ Similarly, another study by Lee et al.^25^ reported 19 patients with refractory CD who received tofacitinib combined with ustekinumab (58%), vedolizumab (37%), and certolizumab pegol (5%). Clinical response and remission were observed in 80% and 60%, respectively. Endoscopic improvement and remission occurred in 54% and 36% of patients, respectively. The outcomes of these open-label combined therapy reports must be viewed in the context of enrolling patients who had failed multiple advanced therapies already.^25^

### Systematic review and meta-analysis

In a meta-analysis published in 2021 by Ahmed et al.,^28^ 30 studies of dual biologic or small-molecule therapy in 279 IBD patients were included (76% CD; median duration of treatment was 24 weeks). The most common dual biologic therapies were anti-TNFs and vedolizumab (48%), ustekinumab and vedolizumab (19%), and 61% of patients had previously failed at least one of the two therapies used in combination. Over a median follow-up of 32 weeks (IQR 24–5.2), the pooled rates of clinical remission and endoscopic remission were 59% (95% CI: 42%–74%) and 34% (95% CI: 23%–46%), respectively. The pooled rates of adverse events (AEs) and serious adverse events(SAEs) were 31% (95% CI: 13%–54%) and 6.5% (95% CI: 2.1%–13.1%), respectively. The proportions of patients who experienced infections and malignancy were 19% (*n* = 52 of 281) and 1% (*n* = 2 of 279), respectively.^28^

A recent meta-analysis by Alayo et al.^29^ in 2022 included 13 studies of 266 patients. The majority of combination therapies were vedolizumab with anti-TNFs (n = 56) or tofacitinib (n = 57). The pooled clinical response and remission rates among patients on vedolizumab/anti-TNF were 77.9% (95% CI: 51.3–97.2) and 55.1% (95% CI: 19.6–88.5), respectively. Among patients on vedolizumab plus tofacitinib, the pooled clinical response and remission rates were 59.9% (95% CI: 37.2–80.8) and 47.8% (95% CI: 19.0–77.4), respectively. With the vedolizumab plus ustekinumab combination, pooled clinical response and remission rates were 83.9% (95% CI: 66.4–96.8) and 47.0% (95% CI: 14.5–80.7), respectively. The pooled endoscopic/radiologic response and remission rates among patients on vedolizumab plus anti-TNF were 38.2% (95% CI: 19.5–58.4) and 18.0% (95% CI: 1.6–41.8), respectively. The corresponding rates among patients on tofacitinib plus vedolizumab were 46.2% (95% CI: 20.4–73.0) and 24.6% (95% CI: 6.4–47.6).^29^

## Clinical implications of combined targeted therapy in IBD management

The rationale for initiation of combined biologic or small-molecule therapy for treatment of complex IBD has been proposed by Privitera et al.^30^ in two clinical scenarios. First, “complicated IBD patients” with poorly controlled luminal disease, where initial co-induction or adding a second agent as sequential induction can be used in case of partial or inadequate response to the first agent. In addition, in high-risk patients who were exposed to multiple biological agents, induction therapy with two agents at the same time may be used as concomitant induction.^30^

**Figure 1.**
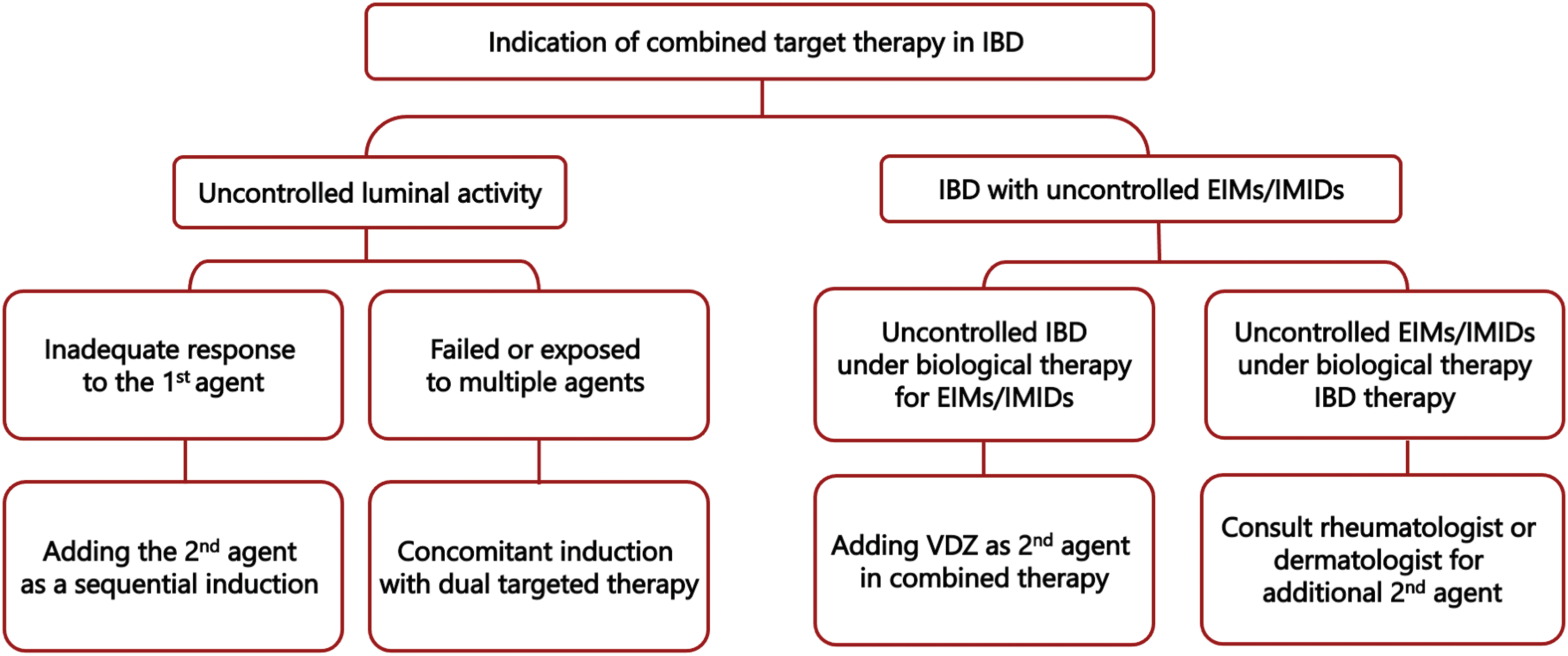
The practical guidance for combined targeted therapy in IBD, adapted from Privitera et al.^30^

The second scenario, “double indication”, involves patients who have concomitant EIMs or IMIDs, where the second agent can be added to control active intestinal inflammation or EIM symptoms.^30^ In IBD patients with uncontrolled luminal symptoms and quiescent EIM symptoms.^30^ The combination of a gut-selective agent, vedolizumab with ustekinumab or anti-TNFs is the most commonly used, followed by ustekinumab with anti-TNFs in the double indication.^20,23,31–33^ The practical guidance for combined targeted therapy in IBD is shown in fig. 1.

There is currently insufficient evidence to suggest routine combination biological or small-molecule therapy in patients with refractory disease or EIMs. Most evidence comes from case reports, case series, retrospective cohorts, and meta-analyses of observational studies where patients were exposed to or refractory to one or both agents used as combination therapy. Thus, the data on combination targeted therapy are restricted by the low level of evidence. Uncontrolled luminal activity and double indications with co-existing EIMs or IMIDs are the two clinical scenarios for starting combination targeted therapy, but more data are needed to better evaluate the benefit-to-risk ratio.

## Safety concerns and adverse events of combined targeted therapy

A major concern of combining biological therapies/small molecules is a possible worsening safety and AEs profile, particularly an increased risk for serious infections or malignancy.^10^ The majority of the data on combined biological therapy come from the rheumatologic literature. A meta-analysis by Boleto et al.,^11^ which included a total of 623 rheumatoid arthritis (RA) patients (410 on combined biologic therapy and 213 on single biologic) with a median follow-up of 9.5 months, suggested that combined biologic therapy in RA appeared to increase the risk of SAEs (14.9% vs. 6.0%).^11^

However, combined biologic or small-molecule therapy in the treatment of IBD seems to have a safer profile and fewer reported SAEs. In a meta-analysis by Ahmed et al,^28^ the proportion of patients or trials that experienced AEs and SAEs was 35% and 6%, respectively. Of those, infections and malignancies accounted for 19% and 1%, respectively.^28^ Of note, a recent meta-analysis by Alayo et al.^29^ in 2022 reported SAEs regarding specific combination types; the pooled rates of SAEs for these were 12.3% for vedolizumab and ustekinumab, 9.6% for vedolizumab and anti-TNF, and 1.0% (95% CI, 0.0–7.6) for tofacitinib and vedolizumab.^29^ In the combination of guselkumab and golimumab studies there was no new single safety concern, One case of tuberculosis was reported in the combination therapy group, and one case of colon adenocarcinoma was reported in the guselkumab monotherapy group.^14^

Thus, the safety of combined biological or small-molecule therapy appears to be acceptable so far, but more data are needed due to limited patient numbers and short-term follow-up.

## Conclusion

The use of combined biological or small-molecule therapies with different targets for the treatment of IBD patients opens a new avenue for the treatment of IBD. “Complex refractory IBD,” where no monotherapeutic option was efficacious, and “double indication,” with active EIMs, are the two clinical scenarios where evidence is accumulating on combined target therapy.^26^ At present, combined targeted therapy cannot be recommended for bio-naïve IBD patients outside of a clinical trial setting. It is unclear whether combination therapy should be used only for the induction period or as maintenance therapy. Furthermore, the optimal combination agents or optimal mechanisms of action for the agents used in combination have not been clearly identified.

Recent findings from the VEGA trial indicated that the combination of novel biologics (guselkumab and golimumab) may become a new treatment option for high-risk UC patients.^14^ The ongoing DUET-CD and DUET-UC trials investigating combining guselkumab and golimumab in refractory IBD patients, are likely to provide additional data on the efficacy of guselkumab and golimumab in CD and UC patients who have failed biologic therapy. However, the treatment armamentarium for IBD is expanding, with the recent approval of multiple non-TNF biologics and small molecules.^34^ In the near future, the combination of these novel agents may also be investigated in clinical trials or in the real-world practice. A further concern of combined targeted therapy is the possible change in safety signals. Although safety seems to be unchanged so far, the data come from small studies with a limited follow-up period. Further large prospective studies with longer follow-up are required to confirm the efficacy and safety of this strategy.

## Data Availability

Not applicable.
